# Non-gravid uterine torsion associated with small bowel obstruction

**DOI:** 10.1093/jscr/rjae045

**Published:** 2024-02-13

**Authors:** Goh Barnabas, Samuel Mathew, Maryam Shamassi, Mohammad Rafique

**Affiliations:** Department of Surgery, Western Health, Melbourne, Victoria 3011, Australia; Department Obstetrics and Gynaecology, Western Health, Melbourne, Victoria 3011, Australia; Department of Anatomical Pathology, Western Health, Melbourne, Victoria 3011, Australia; Department of Surgery, Western Health, Melbourne, Victoria 3011, Australia

**Keywords:** uterine torsion, leiomyoma, bowel obstruction

## Abstract

Uterine torsion is a rare condition. Even more so in cases of non-gravid torsion. We present the case of a post-menopausal woman in her 70s who arrived to our emergency department acutely unwell with abdominal pain and vomiting on a background of a large leiomyomatous uterus, complicated by aspiration pneumonia, acute anaemia, and acute kidney injury. Computed tomography demonstrated a small bowel obstruction secondary to a large heterogeneous calcified pelvic mass. Laparotomy performed demonstrated a large leimyomatous uterus that had torted on the cervical pedicle associated with perforation of the lower anterior segment. A short segment of healthy jejunum was adhered to the uterine fundus, which was easily mobilized. Total hysterectomy and bilateral oophorectomy was performed. The patient made a full recovery. Histopathology demonstrated a calcified leiomyomatous uterus with adjacent haemorrhagic infarction of the uterine wall.

## Introduction

Uterine torsion, whilst common in cattle and buffalo, is an uncommon condition in humans. It is defined as a ˃45° rotation around the long axis, and occurs at the region of the isthmus, which is fixed by the cardinal and uterosacral ligaments [1, 2]. The most common cause of uterine torsion relates to pregnancy, due to an enlarged uterine size and elongation of the isthmus during pregnancy or abnormal foetal presentation [1, 2]. We present a rare case of a small bowel obstruction associated with non-gravid uterine torsion.

## Case report

The patient was a post-menopausal female in her 70s who was brought in via ambulance to the emergency department with 4 days of sudden onset abdominal pain, bilious vomiting, and obstipation. The abdominal pain presented spontaneously, following a bowel motion, and had worsened significantly over 4 days. She had presented to our emergency department on day 1, however as her symptoms had improved, she was subsequently discharged.

Her past history was notable for a large palpable multi fibroid uterus that was only diagnosed 3 months prior on computed tomography (CT) following a presentation to the emergency department with an episode of self-resolving lower abdominal pain—for which she was due to see a gynaecologist. Aside from hypertension, she had no past surgical history. Menarche was at 11 years of age, with menopause at the age of 51. She denied prior history of irregular or heavy periods. She had a normal vaginal delivery at the age of 24.

She presented in respiratory distress, with associated tachycardia and hypotension on arrival. Examination revealed a tender and palpable abdominal mass arising the pelvic region and extending midway between the xiphisternum and umbilicus, the remainder of the abdomen was soft.

Initial venous blood gas noted a metabolic acidosis with a pH of 7.21, bicarbonate of 18 mmol/L, base excess of −6.5, and lactate of 8.3 mmol/L. A haemoglobin of 83 g/DL was noted, a 30 g/DL drop from 4 days prior on her prior presentation. A rise in her white cell count of 13.3 × 10^9^/L and C-reactive protein of 332 mg/L was also noted. Furthermore, she had evidence of a new acute kidney injury with creatinine of 245 mmol/L, urea of 17.3 mmol/L, and glomerular filtration rate of 16 mL/min/1.73m^2^. The patient was fluid resuscitated, a nasogastric tube was inserted with good effect, draining 3.2 L, IV antibiotics were commenced, and an indwelling catheter was inserted.

**Figure 1:**
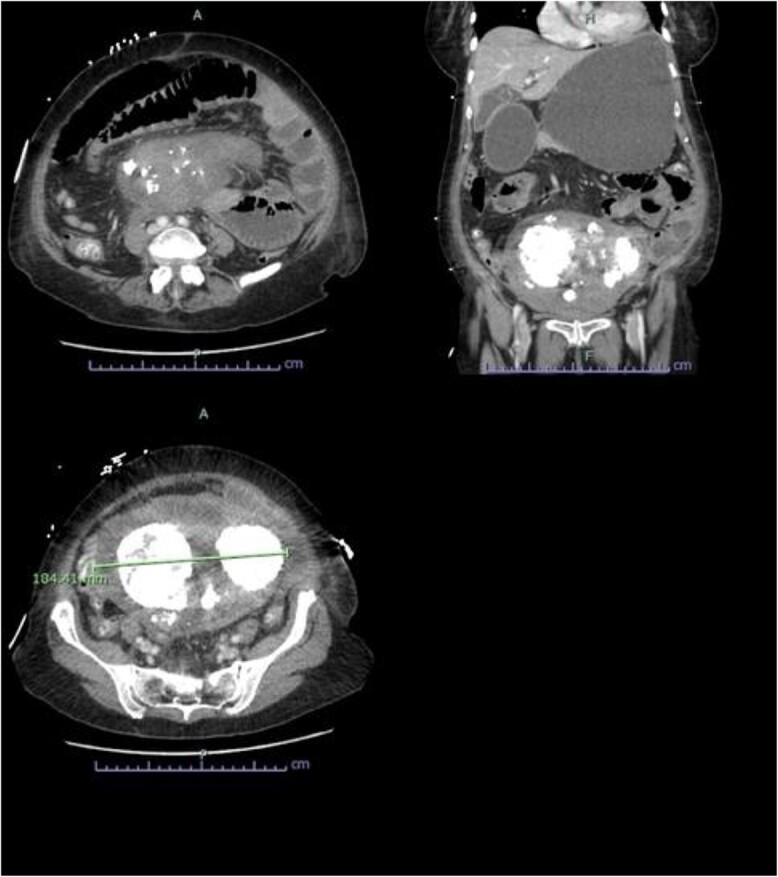
Abdominopelvic CT findings of a large multifibroid uterus with amorphous central calcification associated with a small bowel obstruction.

An abdominopelvic CT was performed, which demonstrated evidence of a small bowel obstruction with evidence of extrinsic compression by a gravid uterus at the level of the duodenojejunal flexure (Fig. 1). The mesentery adjacent to the loop of jejunum was associated with mild stranding, however there was no evidence of pneumatosis, or portal venous gas. Bilateral aspiration pneumonia was also demonstrated on CT.

A surgical consult was obtained due to concerns of bowel ischaemia in the setting of high lactate in conjunction with a gynaecology consult. Whilst the aetiology was unclear, operative intervention was deemed necessary in order to resolve the small bowel obstruction. Given the increasing respiratory support, and acute kidney injury, multidisciplinary consensus on timing led to a decision to proceed with a laparotomy, as further delay would have resulted in increased morbidity and mortality. Following resuscitation with blood products, she was consented for laparotomy.

**Figure 2:**
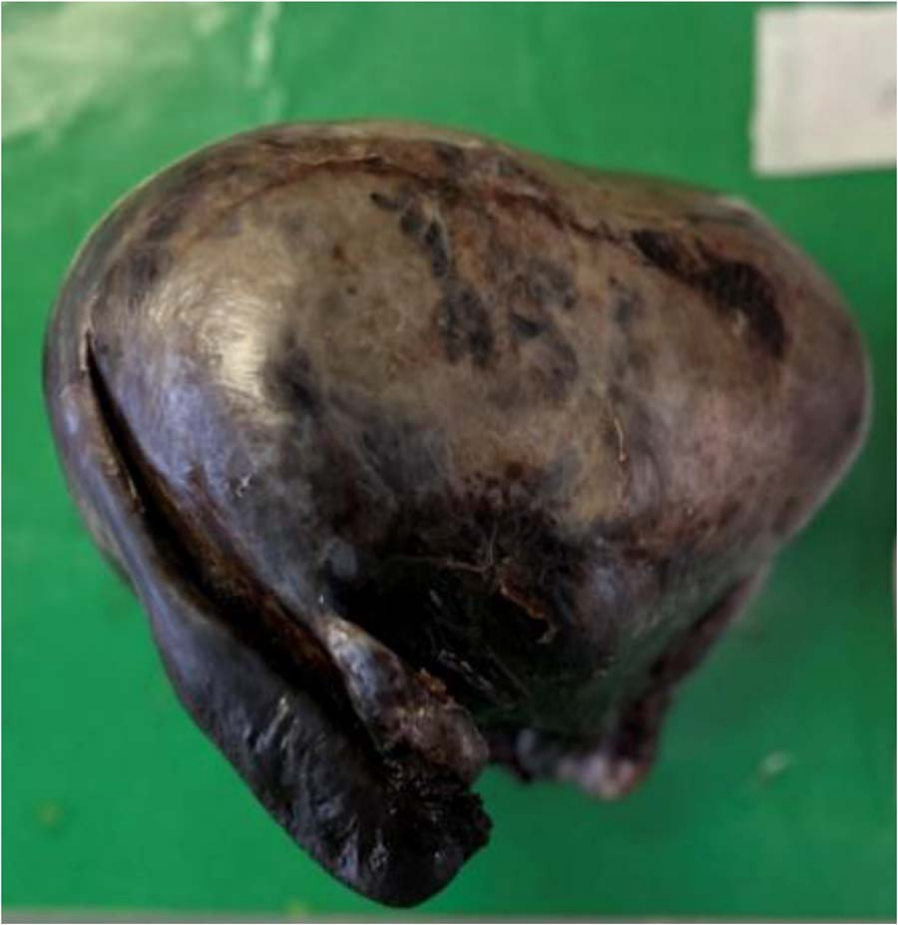
Macroscopic photo of the uterus demonstrating haemorrhage and necrosis (discoloration), in keeping with infarction.

**Figure 3:**
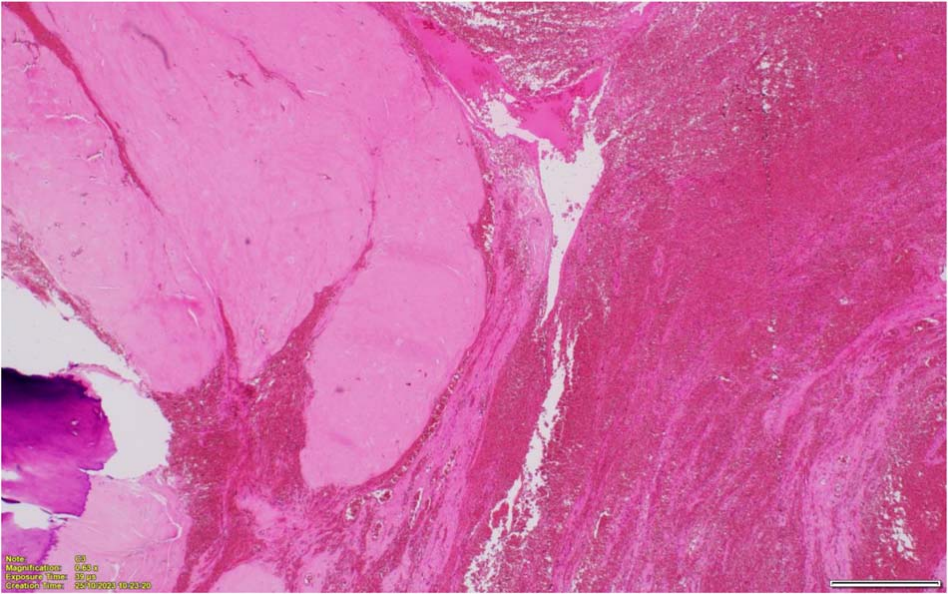
Hyalinized and calcified leiomyoma with adjacent haemorrhagic infarction of the uterine wall, in keeping with torsion.

A midline laparotomy was performed with both members of general surgery and gynaecology present. Findings were notable for a bulky uterus that had torted 1080° on the cervical pedicle. This was associated with a perforated uterus at the anterior lower segment that was actively bleeding. A small portion of small healthy bowel was adhered to the uterus, however there were no evidence of widespread intra-abdominal adhesions. There was no evidence of malignancy. A pinpoint cervix was noted with no evidence of vaginal bleed. Blunt dissection freed the uterus from the small bowel and the uterus was detorted. Ligasure dissection was performed of the bilateral infundibulopelvic ligament ligaments, and uterus amputated above the cervix for haemostasis. A completion total hysterectomy and bilateral salpingo-oophrectomy was then performed. The patient was extubated 2 days post laparotomy, and stepped down to the ward. Both nasogastric tube and in dwelling catheter were removed on day 3 post laparotomy with resumption of bowel function. The patient was discharged on day 8 post laparotomy with ongoing nutritional supplementation. Histopathology demonstrated hyalinized and calcified leiomyoma with adjacent haemorrhagic infarction of the uterine wall, in keeping with torsion (Figs 2 and 3). Subsequently, in her 8 week follow up, she has done markedly well, and has returned to pre-morbid functioning.

## Discussion

There have only been 36 cases of non-gravid uterine torsion documented in adult females since 1980. The most common aetiology relate to uterine leiomyoma. Less common causes include ovarian masses most commonly haemorrhagic ovarian cysts, less so cystadenoma, uterine leiomyosarcoma, or broad ligament leiomyoma [3]. There has also been one case of uterine torsion in a non-gravid, non-myomatous uterus [4] Abdominal symptoms are vague and can include abdominal pain, vomiting. Less commonly, menorrhagia, urinary symptoms, or altered bowel habits [3].

Ultrasonography, CT and magnetic resonance imaging (MRI) have all been listed as potential imaging modalities, however CT and MRI are more sensitive and specific—especially in the non-gravid patient. Nicholson initially described the whirl/whorl sign of the lower uterine segment or uterine cervix as an ‘X’ shape rather than an ‘H’ shape [5] This finding has since been demonstrated on both CT [5–11], and MRI [3, 8, 12]. Other findings include a non-enhancing uterine mass/corpus attached to an enhancing cervix [3, 7, 13], or gas in the uterine cavity [4]. Given the rarity of this condition Uterine torsion was only diagnosed pre-operatively in 38.9% of cases even with prior imaging [8].

In the case we present, MRI was not appropriate given the out of hours presentation and renal insufficiency. The whorl sign was not identified on CT in our patient, and the diagnosis of uterine torsion was only made intra-operatively.

Acute drop in haemoglobin has been described in multiple studies [7–9, 14], which has been attributed to uterine congestion and enlargement in the context of torsion, leading to severe progressive anaemia [3]. Other less frequent complications which were present in our patient included acute kidney injury [14], pneumonia [14]. In our case, another cause of pre-renal acute kidney injury is likely due to persistent vomiting, but also from uterine perforation, which caused a small intra-abdominal bleed. Bowel obstruction has only been noted on one other case of non-gravid bowel obstruction [4].

The most common procedure is total hysterectomy and bilateral salpingo-oophrectomy, however other procedures include partial hysterectomy, and myomectomy [3]—especially where preservation of fertility is required.

In summary, non-gravid bowel obstruction is an uncommon diagnosis that requires a high degree of clinical suspicion. Cross sectional imaging may prove useful in confirming the diagnosis. Given the rarity of the diagnosis, this case study adds valuable information in aiding clinicians when considering the differential for abdominal pain and shock in the context of an enlarged uterus.
